# Longitudinal trajectories of long COVID among hospitalized patients with omicron infection in Changzhi, China

**DOI:** 10.3389/fmed.2026.1867430

**Published:** 2026-07-28

**Authors:** Jianzhou Yang, Jingjing Li, Yuan Li, Liping Yuan, Yuxuan Xue, Tong Wang, Xingyun Xu, Yi Zhu, Zhuo’Ao Zhang, Zhen Qin, Guihua Zhuang, Xiaofeng He

**Affiliations:** 1 School of Public Health, Changzhi Medical College, Changzhi, China; 2 Department of Epidemiology and Biostatistics, School of Public Health, Xi’an Jiaotong University Health Science Center, Xi'an, Shaanxi, China; 3 Institute of Evidence-Based Medicine, Heping Hospital Affiliated to Changzhi Medical College, Changzhi, China; 4 Foshan Center for Disease Control and Prevention, Foshan, Guangdong, China; 5 Health and Wellness Commission of Fanchang District, Wuhu, Anhui, China; 6 The First Department of Clinical Medicine, Changzhi Medical College, Changzhi, China

**Keywords:** COVID-19, follow-up study, long COVID, omicron variant, post-acute COVID-19 syndrome

## Abstract

**Background:**

Understanding the persistence of long COVID (LC) among discharged patients with Omicron infection remains limited.

**Methods:**

The study used a retrospective longitudinal cohort to evaluate the dynamic trajectory of LC after hospital discharge and to identify factors associated with new-onset and persistent LC following Omicron infection. Patients admitted to Heping Hospital Affiliated to Changzhi Medical College and Changzhi People’s Hospital for coronavirus disease 2019 between 15 December 2022 and 30 April 2024 were included. The first follow-up was conducted from 18 July to 20 August 2024, and the second from 9 May 2025 to 13 June 2025.

**Results:**

Of the 3,777 discharged patients, 1,922 [median (IQR) age, 70.0 (59.0–78.0) years; 968 male individuals (50.4%)] who completed both follow-up visits were included in the final analysis. The median (IQR) time from discharge to the second follow-up was 727 (688–854) days. At the second follow-up, 292 patients (15.2%) were diagnosed with LC, including 121 (6.3%) with persistent LC and 171 (8.9%) with new-onset LC. The most common symptoms were muscle weakness, sleep difficulties, fatigue, cough, and dyspnea at rest. Notably, the proportions of muscle weakness, sleep difficulties, cough, arthralgia, and palpitations increased significantly, whereas brain fog decreased significantly from 2.8% at the first follow-up to 0.4% at the second follow-up. Overall, antiviral treatment (OR = 1.503; 95% CI: 1.063–2.126), female sex (OR = 3.729; 95% CI: 1.145–12.147), being a farmer as an occupation (OR = 4.695; 95% CI: 2.188–10.101), and BMI < 18.5 kg/m^2^ (OR = 5.291; 95% CI: 1.115–25.000) were associated with increased new-onset LC susceptibility, whereas having one or more complications was associated with decreased new-onset LC susceptibility (OR = 0.329; 95% CI: 0.125–0.865).

**Conclusion:**

Omicron-infected patients report a persistent burden of symptoms consistent with the WHO definition of LC after discharge. These findings provide valuable information on the dynamic trajectory of long-term health outcomes in patients infected with Omicron. However, the non-specific nature of the symptoms should be considered when interpreting these findings.

## Introduction

Severe acute respiratory syndrome coronavirus 2 (SARS-CoV-2), the causative agent of coronavirus disease 2019 (COVID-19), has evolved into various sub-variants and serotypes (1, 2). Some individuals recovering from acute COVID-19 develop sequelae with persistent symptoms lasting months to years, known as long COVID (LC), post-COVID-19 condition (PCC), or post-COVID-19 syndrome (PCS) (3, 4), which has become a significant public health concern (5, 6). The World Health Organization (WHO) defines LC as a condition occurring in individuals with a history of probable or confirmed SARS-CoV-2 infection, typically developing 3 months after symptom onset, persisting for at least 2 months, and that cannot be explained by an alternative diagnosis (7). Importantly, the WHO definition explicitly notes that symptoms may be new-onset following initial recovery from an acute episode, or may persist from the initial illness, and may fluctuate or relapse over time (7). This definition underscores that LC is not a static condition but can evolve dynamically, including the emergence of symptoms after a period of apparent recovery. These individuals are at high risk of sustained health damage related to declines in physical function and health-related quality of life (8–10). Therefore, systematic follow-up of discharged COVID-19 patients is necessary to identify the dynamic trajectory of LC and to understand the long-term health outcomes of this disease.

Previous studies (11–14) have shown that a substantial proportion of discharged COVID-19 patients continue to experience problems across various health domains. Several meta-analyses have indicated that approximately 30–50% of individuals recovering from SARS-CoV-2 infection develop persistent symptoms lasting up to 1 year (15, 16). Moreover, a meta-analysis showed that, 2 years after the initial spread of COVID-19, up to 42% of infected patients experienced LC (17). However, it is noteworthy that current studies on LC are primarily from Europe and the USA, while studies from Asia are relatively scarce. A meta-analysis observed that studies from the South-East Asia region accounted for only 4.4% of the total (18). Several cohort studies have confirmed that the risk of LC is lower for Omicron than for Delta (19, 20); however, a notable proportion of hospitalized patients continue to experience persistent symptoms (19). PubMed was searched using the keywords “Long COVID” and “China” for studies published on or after 20 November 2025. In total, 592 studies were found, of which 187 were reviews, systematic reviews/meta-analyses, or books and documents. The remaining studies fell into two categories. The first category primarily investigated the pathogenesis, abnormal molecular markers, or genetic factors associated with LC. The second category evaluated risk factors for LC, although the samples were primarily concentrated in Shanghai, Beijing; Guangzhou, Changchun; and Hong Kong, China. Of these, only 10 studies (21–30) analyzed the prevalence and risk factors of LC after Omicron variant infection in China, with only two incorporating two rounds of longitudinal follow-up (22, 29). Moreover, a majority of the samples in these studies were from infections caused by SARS-CoV-2 sub-variants prevalent before 2022 (e.g., BA.1 and BA.2), which cannot reflect LC in China after the epidemic waves caused by subtypes such as BA.4, BA.5, BF.7, BQ.1, and XBB. Whether COVID-19-related symptoms may persist for a longer duration remains an open question. In China, only one previous study (31) evaluated the health status of COVID-19 survivors who had been hospitalized for more than 2 years and identified factors associated with an increased risk of persistent symptoms, but that study focused on patients infected with the original strain who were hospitalized in Wuhan in early 2020.

To the best knowledge of the authors, this is one of the few longitudinal cohort studies with two waves of follow-up (up to > 2 years after discharge) focusing exclusively on hospitalized Omicron-infected patients in China, providing unique insights into the dynamic trajectory of LC beyond the first year. Recent international longitudinal studies have also highlighted the changing prevalence of LC across different variants and populations (19, 20, 29, 32–35). In this study, a longitudinal cohort design was applied to systematically assess the dynamic trajectory of LC and the relevant influencing factors for persistent LC symptoms after discharge among Omicron-infected patients in Changzhi, China.

## Methods

### Study design and participants

**Figure 1:**
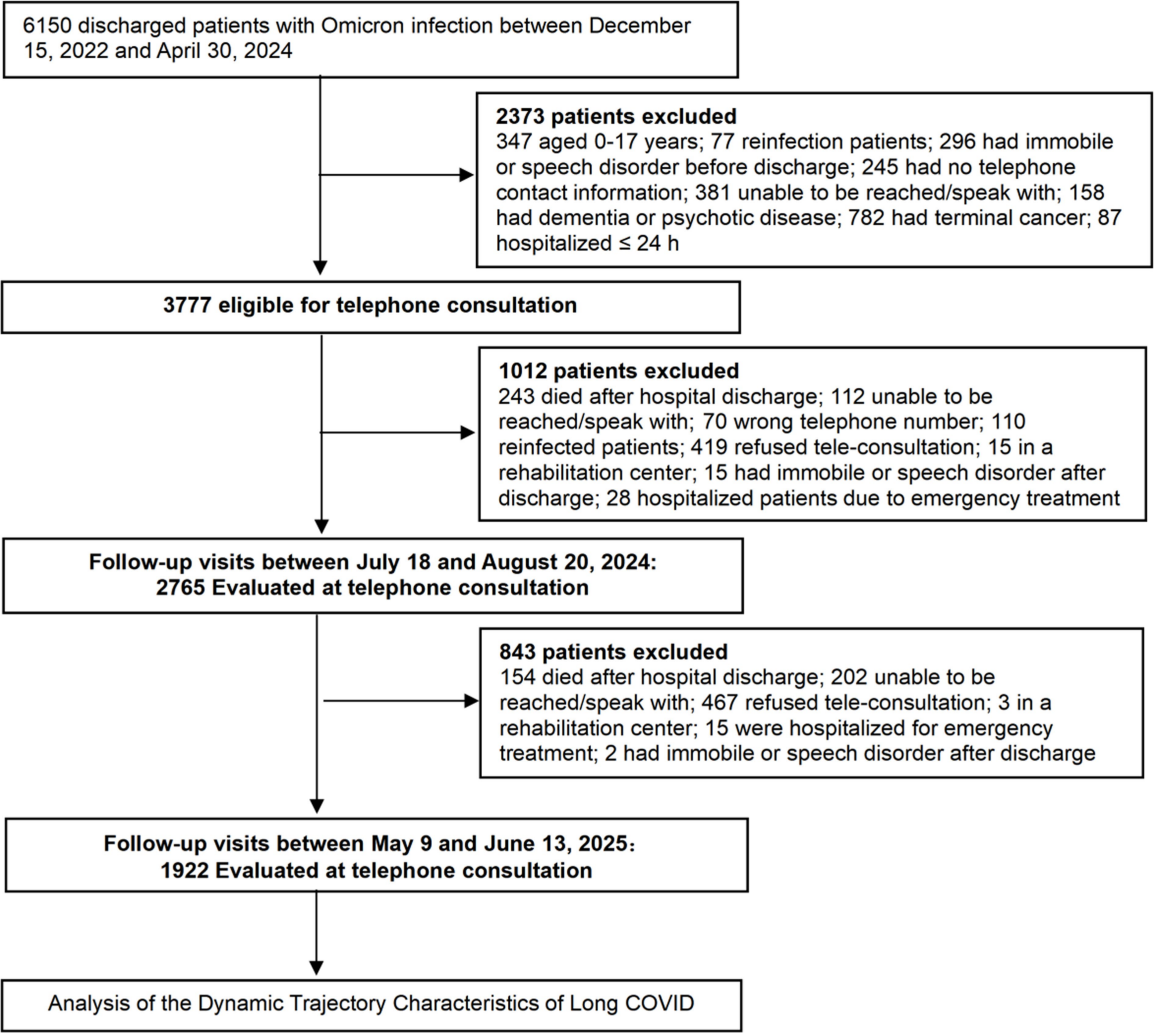
Flow of patient screening and enrollment.

This retrospective longitudinal cohort study included patients admitted to Heping Hospital Affiliated to Changzhi Medical College and Changzhi People’s Hospital in China for COVID-19 between 15 December 2022 and 30 April 2024. The inclusion criteria were as follows: (1) age ≥ 18 years, (2) hospitalization for SARS-CoV-2 infection, (3) hospital stay of at least 24 h, and (4) diagnosis of SARS-CoV-2 infection confirmed by RT-PCR assay in all cases; chest CT findings were used as supplementary evidence and were not used as the sole diagnostic criterion. The exclusion criteria were as follows: (1) death during follow-up after discharge, (2) long-term hospitalization, (3) placement in a nursing or welfare home after discharge, (4) terminal cancer, (5) nosocomial SARS-CoV-2 infection, (6) psychotic disorder or dementia, (7) speech disorder, and (8) SARS-CoV-2 reinfection, defined as a new positive RT-PCR test or the new onset of typical COVID-19 symptoms with epidemiological linkage occurring at least 90 days after the initial discharge, as identified through patient self-report during follow-up interviews and cross-checked with hospital readmission records where available (36). Reinfection status was assessed using structured patient self-report during follow-up interviews, supplemented by cross-checking against hospital readmission records for those who were re-hospitalized. Based on the above criteria, a total of 3,777 discharged COVID-19 patients with Omicron infection were eligible and contacted via telephone (Figure 1). In this telephone-based follow-up study, baseline data were collected only after patients consented to participate in the follow-up interview. For patients who were excluded (e.g., those who died, refused, or were lost to contact), systematic baseline data could not be retrieved, thus precluding a formal comparison between included and excluded patients. This study was conducted in accordance with the Declaration of Helsinki. The study protocol was approved by the Ethics Committee of Heping Hospital Affiliated to Changzhi Medical College [approval number (2024)087]. Baseline and follow-up data were collected only after participants provided verbal informed consent via telephone. The ethics committee specifically approved this consent procedure for the telephone-based follow-up design. All data were anonymized prior to analysis. This study is reported according to the Strengthening the Reporting of Observational Studies in Epidemiology (STROBE) reporting guideline (Supplementary material).

### Definitions of LC

LC was defined according to the WHO as a condition occurring in individuals with a history of probable or confirmed SARS-CoV-2 infection, typically developing three months after symptom onset, persisting for at least 2 months, and that cannot be explained by an alternative diagnosis (7). This WHO definition served as the primary case definition. In this study, we evaluated LC symptoms using a pre-designed questionnaire (Supplementary Table S1) covering fatigue, muscle weakness, sleep difficulties, and others. During follow-up via the telephone, we systematically inquired symptom duration to confirm persistence for at least 2 months and actively asked about preexisting or intercurrent illnesses that might assist in excluding alternative causes; the responses were cross-checked against available medical records. In addition, the patients were systematically asked whether each symptom had existed prior to their SARS-CoV-2 infection. Individuals with at least one of these symptoms meeting the WHO criteria were classified as having LC. Based on the dynamic changes in the number of symptoms between the two follow-up visits, individuals were divided into four categories: (1) persistent LC—at least one symptom at both follow-ups; (2) resolved LC—at least one symptom at the first follow-up but none at the second; (3) new-onset LC—no symptoms at the first follow-up but at least one symptom at the second. This category aligns with the WHO definition (7), which allows for symptoms to be new-onset after initial recovery, and has been observed in large cohorts such as RECOVER (32); and (4) no symptoms—asymptomatic at both follow-ups.

### Telephone assessment and data acquisition

Patients were contacted via telephone by one infectious disease physician and six trained public health and preventive medicine students at appropriate time points after discharge using a structured questionnaire (Supplementary Table S1) to investigate LC symptoms. The first follow-up was conducted between 18 July 2024 and 20 August 2024 after patient discharge (Figure 1). The second follow-up was conducted between 9 May 2025 and 13 June 2025 (Figure 1). During the telephone follow-up, patients were asked to confirm whether they had previous or recent illnesses causing symptoms, such as self-limiting illnesses (e.g., influenza) or chronic respiratory diseases (e.g., chronic obstructive pulmonary disease). Information provided by the patient was reviewed before the end of the call to confirm accuracy and to check for omissions or additions. At the end of each round of follow-up, approximately 10% of patients were randomly selected for a second telephone interview to verify information consistency, which is a commonly used quality control approach in large-scale telephone surveys (the proportion was chosen based on operational feasibility rather than statistical power calculation) (37), and the median values from the two assessments was used.

This study retrospectively collected comprehensive baseline data from hospitalized COVID-19 patients through the hospital’s medical record system, with supplementary information obtained during follow-up visits to address missing details. The data included demographic and clinical parameters, such as age, sex, height, weight, smoking history, occupation, admission and discharge dates, and a range of comorbidities. The study also documented the use of antiviral drugs and immunosuppressants, mechanical ventilation requirements, and major symptoms during the acute exacerbation phase. Severe and non-severe patients was classified according to the Guidelines for Diagnosis and Treatment of SARS-CoV-2 Infection (version 10) issued by the National Health Commission of China (38). COVID-19 vaccination status was evaluated based on self-reported data; a majority of the vaccinated patients reported receiving an inactivated COVID-19 vaccine. They were classified as unvaccinated, vaccinated with one dose, vaccinated with two doses, or vaccinated with three doses.

### Outcome definition

The primary outcome of this study was the prevalence and dynamic trajectory of LC at the second follow-up visit. Secondary outcomes included changes in individual symptom proportions between the two follow-ups and the identification of influencing factors (demographic, clinical, and occupational) associated with new-onset and persistent LC.

### Statistical analysis

The minimum required sample size was estimated as 433 discharged patients using the formula: *N* = (*Z*1-*α*/2/*δ*)2 × *p* × (1–*p*). The confidence interval (CI) was set at 95%, the expected prevalence of LC was 8.9% (22), the margin of error was set at 3% (39), and the loss to follow-up rate was assumed to be 20%. Baseline characteristics were presented as counts (percentages) for categorical variables and median [interquartile range (IQR)] for continuous variables. Differences in categorical variables were compared using the chi-squared test or Fisher’s exact test. Differences in continuous variables were compared using the Mann–Whitney *U* test. Univariate and multivariate logistic regression analyses were used to investigate potential factors influencing LC. The crude odds ratio (OR) was derived from the univariate analysis, and the adjusted OR (aOR) was obtained from the multivariate analysis. Variables with *a p-*value of < 0.2 in the univariable analysis were included in the multivariable analysis. There were no missing data for the baseline or follow-up variables; all analyses were based on the complete dataset of 1,922 patients. Moreover, for the main multivariable models, the events-per-variable (EPV) ratios were as follows: (1) for new-onset LC, 171 events with 4 covariates yielded an EPV of approximately 42.8:1; for persistent LC, 121 events with 5 covariates yielded an EPV of approximately 24.2:1. Both ratios substantially exceeded the recommended minimum of 10:1, indicating adequate statistical power and a low risk of overfitting. Collinearity was assessed using variance inflation factors (VIFs), and all VIFs were < 2.0, indicating no significant multicollinearity among the included covariates. In addition to univariable screening (*p* < 0.2), variable selection was guided by clinical relevance, with age, sex, disease severity, and antiviral treatment considered *a priori* based on the established literature. The final models were determined using stepwise forward likelihood-ratio regression, balancing statistical significance with clinical interpretability. No adjustment for multiple comparisons was applied, as subgroup and symptom-level analyses were explicitly exploratory. All tests were two-sided, and *a p-*value of < 0.05 was considered statistically significant. All statistical analyses were performed using IBM SPSS Statistics 25.0.

## Results

### Patient characteristics

Among 6,150 discharged patients with Omicron infection between 15 December 2022 and 30 April 2024, 3,777 patients were screened for eligibility and contacted by telephone. A total of 1,922 patients (50.9%) who completed both follow-ups were included in the final analysis (Figure 1). All 1,922 patients included in the final analysis had RT-PCR-confirmed SARS-CoV-2 infection. Among the 3,777 eligible patients, 1,855 of them (49.1%) were excluded due to their inability to complete both telephone assessment. As detailed in Figure 1, the primary reasons for exclusion included refusal to participate (28.8%), death during follow-up (10.5%), inability to be reached (8.3%), and other predefined criteria. The median (IQR) age of included patients was 70.0 (59.0–78.0) years, with 287 (15.0%) aged 18–49 years, 218 (11.3%) aged 50–59 years, 425 (22.1%) aged 60–69 years, 554 (28.8%) aged 70–79 years, and 438 (22.8%) aged ≥ 80 years. Of these patients, 968 of them (50.4%) were male (Table 1). Moreover, 1,818 discharged patients (94.6%) had at least one comorbidity, mainly hypertension (46.6%), cardiovascular disease (32.5%), and diabetes (23.2%) (Table 1). Furthermore, 516 patients (26.8%) were unvaccinated or had received only one dose of COVID-19 vaccine, while 1,406 (73.2%) had received at least two doses of the vaccine (Table 1). During hospitalization, 1,220 patients (63.5%) received antiviral treatment, 683 patients (35.5%) received immunosuppressant therapy, and 59 patients (3.1%) received mechanical ventilation. Finally, 1,037 patients (54.0%) were categorized as having severe COVID-19. The median (IQR) time from discharge to follow-up was 441 (403–570) days at the first follow-up and 727 (688–854) days at the second follow-up. The median (IQR) duration of hospital stay was 9 (6–12) days. No significant differences were found in BMI, diabetes, cerebrovascular disease, chronic kidney disease, liver disease, autoimmune diseases, organ transplantation, or days of follow-up between severe and non-severe COVID-19 patients (Table 1).

**Table 1:** Demographic and clinical characteristics of participants.

Characteristics ^a^	Severe disease [*N* = 1,037 (54.0)]	Non-severe disease [*N* = 885 (46.0)]	Overall (*N* = 1922)	*p*-value ^b^
Age (years)				
Overall (median, IQR)	73.0 (65.0, 81.0)	65.0 (45.0, 68.0)	70.0 (59.0, 78.0)	< 0.001
18–49	56 (5.4)	231 (26.1)	287 (15.0)	< 0.001
50–59	104 (10.0)	114 (12.9)	218 (11.3)	
60–69	234 (22.6)	191 (21.6)	425 (22.1)	
70–79	339 (32.7)	215 (24.3)	554 (28.8)	
≥ 80	304 (29.3)	134 (15.1)	438 (22.8)	
Sex				
Male	590 (56.9)	378 (42.7)	968 (50.4)	< 0.001
Female	447 (43.1)	507 (57.3)	954 (49.6)	
Occupation				
Farmer	361 (34.8)	232 (26.2)	593 (30.9)	< 0.001
Non-farmer	676 (65.2)	653 (73.8)	1,329 (69.1)	
Vaccination				
Unvaccinated or one dose	305 (29.4)	211 (23.8)	516 (26.8)	0.006
Two or more doses	732 (70.6)	674 (76.2)	1,406 (73.2)	
Number of comorbidities				
0	11 (1.1)	93 (10.5)	104 (5.4)	< 0.001
≥ 1	1,026 (98.9)	792 (89.5)	1818 (94.6)	
Comorbidities				
Hypertension	540 (52.1)	355 (40.1)	895 (46.6)	0.023
Diabetes	248 (23.9)	198 (22.4)	446 (23.2)	0.425
Cardiovascular disease	372 (35.9)	252 (28.5)	624 (32.5)	0.001
Cerebrovascular disease	207 (20.0)	153 (17.3)	360 (18.7)	0.134
Chronic kidney disease	192 (18.5)	141 (15.9)	333 (17.3)	0.136
Neurodegenerative disorder	20 (1.9)	48 (5.4)	68 (3.5)	< 0.001
Liver disease	214 (20.6)	180 (20.3)	394 (20.5)	0.872
Autoimmune diseases	8 (0.8)	9 (1.0)	17 (0.9)	0.567
Thyroid dysfunction	29 (2.8)	62 (7.0)	91 (4.7)	< 0.001
Organ transplantation	6 (0.6)	8 (0.9)	14 (0.7)	0.403
BMI (kg/m2)				
< 18.5	73 (7.0)	49 (5.5)	122 (6.3)	0.224
18.5–23.9	401 (38.7)	373 (42.2)	774 (40.3)	
24–27.9	415 (40.0)	329 (37.2)	744 (38.7)	
≥ 28	148 (14.3)	134 (15.1)	282 (14.7)	
Smoke				
Never smoke	783 (75.5)	721 (81.5)	1,504 (78.3)	0.002
Current/Previous smoke	254 (24.5)	164 (18.5)	418 (21.7)	
Number of acute symptoms				
≤ 2	278 (26.8)	351 (39.7)	629 (32.7)	< 0.001
3–4	478 (46.1)	325 (36.7)	803 (41.8)	
5–6	199 (19.2)	143 (16.2)	342 (17.8)	
≥ 7	82 (7.9)	66 (7.4)	148 (7.7)	
Antiviral treatment				
No	296 (28.5)	406 (45.9)	702 (36.5)	< 0.001
Yes	741 (71.5)	479 (54.1)	1,220 (63.5)	
Immunosuppressant therapy				
No	579 (55.8)	660 (74.6)	1,239 (64.5)	< 0.001
Yes	458 (44.2)	225 (25.4)	683 (35.5)	
Mechanical ventilation				
No	978 (94.3)	885 (100.0)	1863 (96.9)	< 0.001
Yes	59 (5.7)	0 (0.0)	59 (3.1)	
Hospitalization (days)	10.0 (7.0, 13.0)	8.0 (5.0, 11.0)	9.0 (6.0, 12.0)	< 0.001
Follow-up (days)	721.0 (684.0, 851.0)	731.0 (694.0, 858.0)	727.0 (688.0, 854.0)	0.283

IQR, interquartile range; ^a^ Continuous variables were expressed as medians (interquartile ranges), and categorical variables were expressed as frequencies (percentages); ^b^ *p*-values were calculated using rank sum and chi-square tests. Bold p-values were statistically significant, i.e., *p* < 0.05.

### Characteristics of LC symptoms at the first and the second follow-up

**Figure 2:**
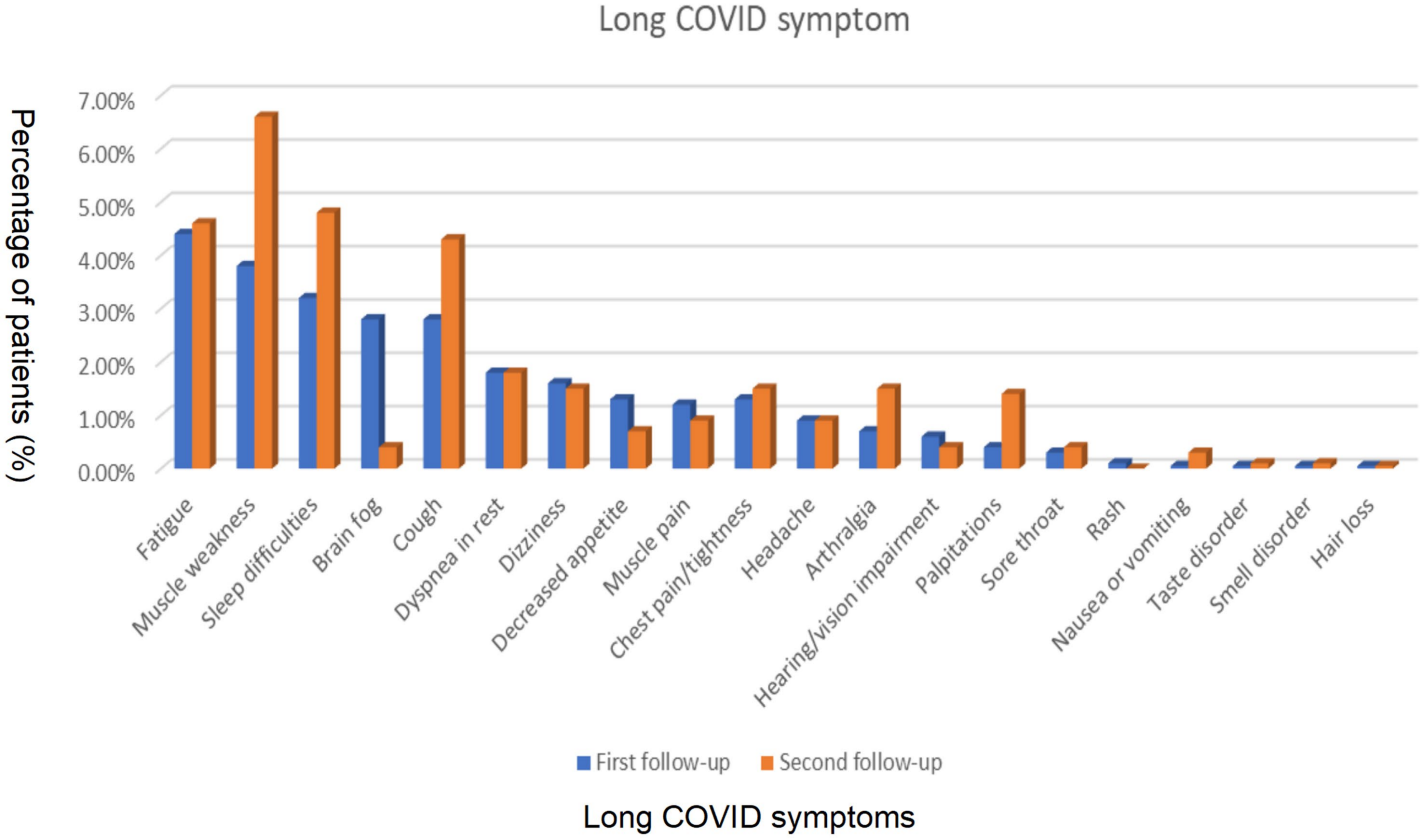
Percentage of patients presenting with long COVID symptoms at the first and the second follow-up.

During hospitalization, 1,884 patients (98.0%) had at least one COVID-19-related symptom. During the overall follow-up period, the proportion of patients with LC did not change significantly [first follow-up vs. second follow-up: 16.3% (313 patients) vs. 15.2% (292 patients); difference, 1.1%; 95% CI, 0.7–1.7%; *p* = 0.352, Table 2]. Among those with symptoms at the second follow-up, 89 patients (4.6%) reported one symptom and 203 (10.6%) reported at least two symptoms. At the first follow-up, the most common LC symptoms were fatigue, muscle weakness, sleep difficulties, brain fog, and cough, whereas at the second follow-up, the most common symptoms were muscle weakness, sleep difficulties, fatigue, cough, and dyspnea at rest (Figure 2; Table 2). At both time points, a majority of the symptoms showed no significant change (Table 2). However, the proportions of muscle weakness, sleep difficulties, cough, arthralgia, and palpitations increased significantly (*p* < 0.05, Table 2), whereas brain fog decreased significantly from 2.8% at the first follow-up to 0.4% at the second follow-up (*p* < 0.05, Table 2).

**Table 2:** Percentage of patients presenting with long COVID related symptoms at the first and second follow-up visits.

	First follow-up (*N* = 1922)	Second follow-up (*N* = 1922)	*P*-value
Number of self-reported LC symptoms			
0	1,609 (83.7)	1,630 (84.8)	0.352
≥ 1	313 (16.3)	292 (15.2)	
Self-reported LC symptoms			
Fatigue	85 (4.4)	89 (4.6)	0.756
Muscle weakness	73 (3.8)	127 (6.6)	**< 0.001**
Sleep difficulties	62 (3.2)	93 (4.8)	**0.011**
Brain fog	53 (2.8)	8 (0.4)	**< 0.001**
Cough	53 (2.8)	84 (4.3)	**0.007**
Dyspnea in rest	34 (1.8)	34 (1.8)	1.000
Dizziness	30 (1.6)	28 (1.5)	0.791
Decreased appetite	25 (1.3)	13 (0.7)	0.050
Muscle pain	23 (1.2)	17 (0.9)	0.340
Chest pain/tightness	25 (1.3)	28 (1.5)	0.678
Headache	17 (0.9)	18 (0.9)	0.865
Arthralgia	13 (0.7)	28 (1.5)	**0.019**
Hearing/vision impairment	12 (0.6)	8 (0.4)	0.370
Palpitations	7 (0.4)	26 (1.4)	**0.001**
Sore throat	5 (0.3)	8 (0.4)	0.405
Rash	2 (0.1)	0 (0.0)	0.096
Nausea or vomiting	1 (0.05)	6 (0.3)	0.125
Taste disorder	1 (0.05)	2 (0.1)	0.560
Smell disorder	1 (0.05)	2 (0.1)	0.560
Hair loss	1 (0.05)	1 (0.05)	1.000

Data are n (%), unless otherwise specified. Bold *P* value show statistically significant, i.e., *P* < 0.05.

### Dynamic trajectory of LC

**Figure 3:**
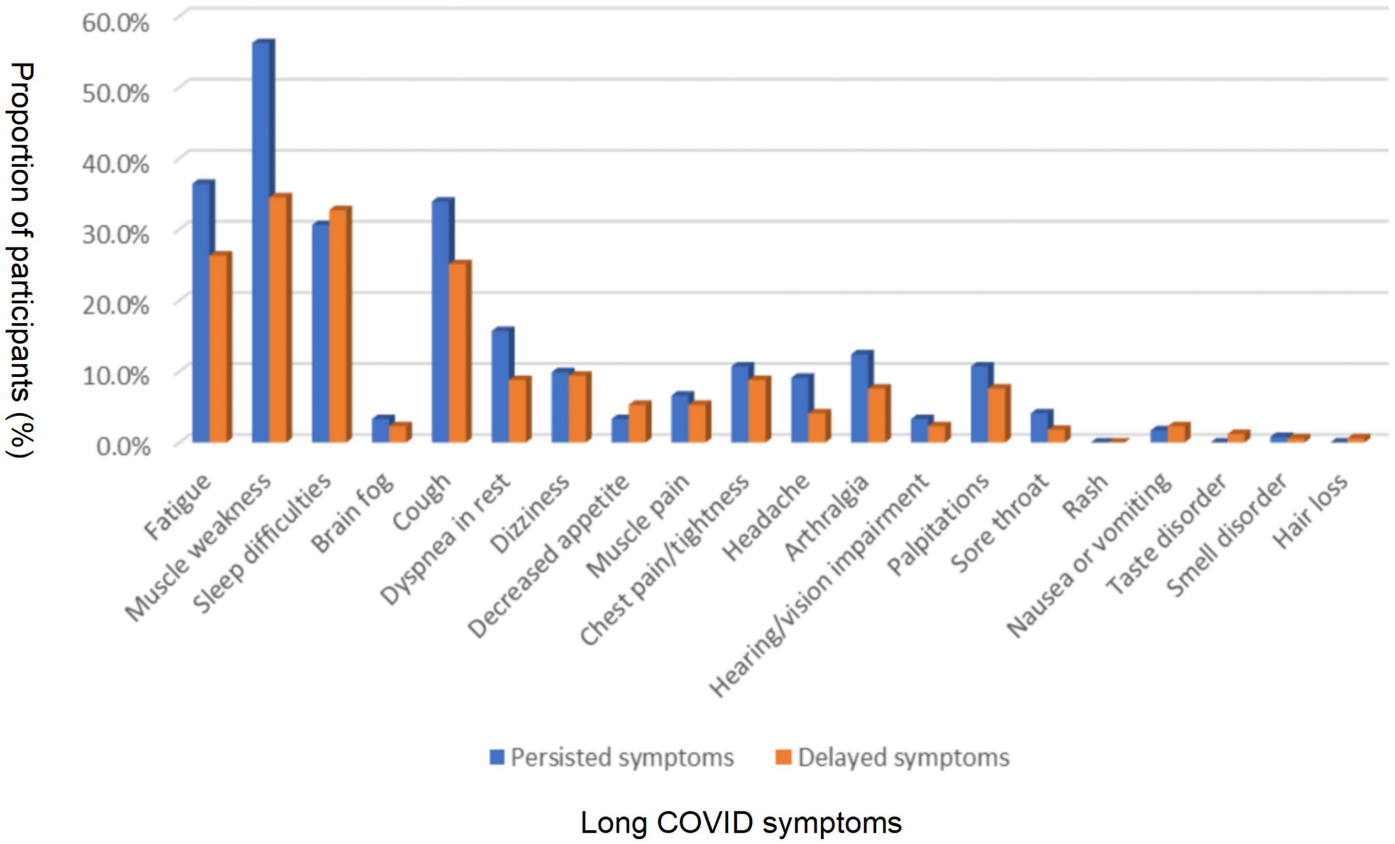
Proportion of persisted and delayed symptoms at the second follow-up.

Regarding the dynamics of LC at the second follow-up, 121 patients (6.3%) were classified as having persistent LC, 192 (10.0%) as resolved LC, 171 (8.9%) as new-onset LC, and 1,438 (74.8%) as having no symptoms (Table 3). Patients with severe COVID-19 were more likely to develop LC compared with those with non-severe patients [18.7% (194 patients) vs. 13.4% (119 patients) at the first follow-up, *p* = 0.002; 16.9% (175 patients) vs. 13.2% (117 patients) at the second follow-up, *p* = 0.026, Table 3]. Patients with severe COVID-19 were also more likely to be classified as having persistent LC [severe vs. non-severe: 7.8% (81 patients) vs. 4.5% (40 patients) at the second follow-up, *p* = 0.003, Table 3] and less likely to be classified as having no symptoms [severe vs. non-severe, 72.2% (749 patients) vs. 77.9% (689 patients), *p* = 0.005, Table 3]. Subgroup analyses based on different follow-up intervals showed similar patterns. For example, when the first follow-up occurred at 360–539 days [14.3 (13.9, 14.6) months] and the second follow-up occurred at 638–826 days [23.7 (23.3, 24.1) months], patients with severe COVID-19 were more likely to develop LC compared with those with non-severe COVID-19 [17.0% (70 patients) vs. 9.6% (31 patients) at the first follow-up, *p* = 0.004, Table 3] and were more likely to have persistent LC [9.0% (37 patients) vs. 3.4% [11 patients], *p* = 0.002, Table 3]. No significant results were observed in other subgroup analyses (Table 3). Muscle weakness, fatigue, cough, sleep difficulties, and dyspnea at rest were the most common symptoms in the persistent LC group (Figure 3). The most common symptoms in the new-onset LC group were muscle weakness, sleep difficulties, fatigue, cough, and dizziness (Figure 3). Notably, the proportions of fatigue (36.4% vs. 26.3%), muscle weakness (56.2% vs. 34.5%), and dyspnea at rest (15.7% vs. 8.8%) were much higher in the persistent LC group than in the delayed LC group.

**Table 3:** Dynamics of long COVID according to disease severity.

Classification	Diagnosis of LC		Patients, No. (%)			*P*-value^a^
	1st follow-up	2nd follow-up	Enrolled patients	Severe disease	Non-severe disease	
Overall Omicron follow-up period						
Total	NA	NA	1922 (100.0)	1,037 (100.0)	885 (100.0)	NA
LC at 1st follow-up	Yes	-	313 (16.3)	194 (18.7)	119 (13.4)	**0.002**
LC at 2nd follow-up	-	Yes	292 (15.2)	175 (16.9)	117 (13.2)	**0.026**
Persistent LC	Yes	Yes	121 (6.3)	81 (7.8)	40 (4.5)	**0.003**
Resolved LC	Yes	No	192 (10.0)	113 (10.9)	79 (8.9)	0.151
Delayed LC	No	Yes	171 (8.9)	94 (9.1)	77 (8.7)	0.780
No symptoms	No	No	1,438 (74.8)	749 (72.2)	689 (77.9)	**0.005**
First follow-up at 90–359 days [7.2 (4.4, 10.3) months] while second follow-up at 375–660 days [17.0 (14.0, 20.1) months]						
Total	NA	NA	382 (100.0)	197 (100.0)	185 (100.0)	NA
LC at 1st follow-up	Yes	-	69 (18.1)	41 (20.8)	28 (15.1)	0.149
LC at 2nd follow-up	-	Yes	52 (13.6)	31 (15.8)	21 (11.4)	0.212
Persistent LC	Yes	Yes	19 (5.0)	11 (5.6)	8 (4.3)	0.572
Resolved LC	Yes	No	50 (13.1)	30 (15.2)	20 (10.8)	0.201
Delayed LC	No	Yes	33 (8.6)	20 (10.2)	13 (7.1)	0.277
No symptoms	No	No	280 (73.3)	136 (69.0)	144 (77.8)	0.052
First follow-up at 360–539 [14.3 (13.9, 14.6)] months while second follow-up at 638–826 days [23.7 (23.3, 24.1) months]						
Total	NA	NA	734 (100.0)	411 (100.0)	323 (100.0)	NA
LC at 1st follow-up	Yes	-	101 (13.7)	70 (17.0)	31 (9.6)	**0.004**
LC at 2nd follow-up	-	Yes	108 (14.7)	67 (16.3)	41 (12.7)	0.171
Persistent LC	Yes	Yes	48 (6.5)	37 (9.0)	11 (3.4)	**0.002**
Resolved LC	Yes	No	53 (7.2)	33 (8.0)	20 (6.2)	0.340
Delayed LC	No	Yes	60 (8.2)	30 (7.3)	30 (9.3)	0.329
No symptoms	No	No	573 (78.1)	311 (75.7)	262 (81.1)	0.077
First follow-up at ≥ 540 days [19.1 (18.8, 19.4)] months while second follow-up at 827—907 days [28.6 (28.3, 28.9) months]						
Total	NA	NA	806 (100.0)	429 (100.0)	377 (100.0)	NA
LC at 1st follow-up	Yes	-	143 (17.7)	83 (19.4)	60 (15.9)	0.203
LC at 2nd follow-up	-	Yes	132 (16.4)	77 (18.0)	55 (14.6)	0.198
Persistent LC	Yes	Yes	54 (6.7)	33 (7.7)	21 (5.6)	0.229
Resolved LC	Yes	No	89 (11.0)	50 (11.7)	39 (10.3)	0.554
Delayed LC	No	Yes	78 (9.7)	44 (10.3)	34 (9.0)	0.553
No symptoms	No	No	585 (72.6)	302 (70.3)	283 (75.1)	0.138

^a^ Bold *P* value show statistically significant, i.e., *P* < 0.05.

### Factors associated with new-onset and persistent LC

Overall, the multivariable analysis showed that antiviral treatment was associated with increased susceptibility to new-onset LC (OR = 1.503; 95% CI: 1.063–2.126; *p* = 0.021, Table 4). In subgroup analyses based on different follow-up times, when the first follow-up visits occurred at 90–359 days [7.2 (4.4, 10.3) months] and the second follow-up visits occurred at 375–660 days [17.0 (14.0, 20.1) months], female sex (OR = 3.729; 95% CI: 1.145–12.147; *p* = 0.029), farmer by occupation (OR = 4.695; 95% CI: 2.188–10.101; *p* < 0.001), and BMI < 18.5 kg/m^2^ (OR = 5.291; 95% CI: 1.115–25.000; *p* = 0.036) were associated with increased susceptibility to new-onset LC (Supplementary Table S2). When the first follow-up was at 360–539 days [14.3(13.9, 14.6) months] and the second follow-up was at 638–826 days [23.7 (23.3, 24.1) months], female sex (OR = 1.971; 95% CI: 1.132–3.431; *p* = 0.017) remained a significantly associated factor (Supplementary Table S3). When the first follow-up was at ≥ 540 days [19.1 (18.8, 19.4) months] and the second follow-up at 827–907 days [28.6 (28.3, 28.9) months], having one or more comorbidities (OR = 0.329; 95% CI: 0.125–0.865; *p* = 0.024) was associated with decreased susceptibility to new-onset LC, whereas antiviral treatment (OR = 2.435; 95% CI: 1.381–4.292; *p* = 0.002) was associated with increased susceptibility (Supplementary Table S4). Subsequently, we investigated factors associated with susceptibility to persistent LC compared with susceptibility to resolved LC. No significant associations were found in the multivariate analysis (Table 5).

**Table 4:** Logistic regression models to evaluate the risk factors for new-onset LC vs. no symptoms during overall Omicron period.

Characteristics ^a^	Total	No symptoms [*n* (%)]	Delayed LC [*n* (%)]	OR (95% CI)	*P*-value	aOR (95% CI)	*P*-value
Overall	1,609	1,438 (89.4)	171 (10.6)	.	.	.	.
Age (years)							
18–49	248	228 (91.9)	20 (8.1)	1 (ref)	.	1 (ref)	.
≥ 50	1,361	1,210 (88.9)	151 (11.1)	1.423 (0.874, 2.316)	0.156	1.197 (0.720, 1.988)	0.488
Gender							
Male	811	730 (90.0)	81 (10.0)	1 (ref)	.	1 (ref)	.
Female	798	708 (88.7)	90 (11.3)	1.146 (0.834, 1.574)	0.401	1.157 (0.839, 1.595)	0.374
Occupation							
Non-farmer	1,127	1,018 (90.3)	109 (9.7)	1 (ref)	.	1 (ref)	.
Farmer	482	420 (87.1)	62 (12.9)	1.379 (0.989, 1.923)	0.058	1.379 (0.989, 1.923)	0.058
Vaccination							
Unvaccinated or one dose	444	400 (90.1)	44 (9.9)	1 (ref)	.	1 (ref)	.
Two or more doses	1,165	1,038 (89.1)	127 (10.9)	1.112 (0.775, 1.597)	0.564	1.156 (0.803, 1.662)	0.436
Number of comorbidities							
0	91	80 (87.9)	11 (12.1)	1 (ref)	.	1 (ref)	.
≥ 1	1,518	1,358 (89.5)	160 (10.5)	0.857 (0.447, 1.643)	0.642	0.660 (0.335, 1.300)	0.230
BMI (kg/m2)							
< 18.5	99	84 (84.8)	15 (15.2)	1.616 (0.881, 2.959)	0.121	.	.
18.5–23.9	653	588 (90.0)	65 (10.0)	1 (ref)	.	1 (ref)	.
24–27.9	621	554 (89.2)	67 (10.8)	1.094 (0.763, 1.569)	0.625	.	.
≥ 28	236	212 (89.8)	24 (10.2)	1.012 (0.791, 1.295)	0.925	1.038 (0.810, 1.331)	0.769
Smoke							
Never smoker	1,255	1,119 (89.2)	136 (10.8)	1 (ref)	.	1 (ref)	.
Current/Previous smoke	354	319 (90.1)	35 (9.9)	0.903 (0.610, 1.336)	0.609	0.909 (0.613, 1.347)	0.634
Number of acute symptoms							
≤ 2	538	475 (88.3)	63 (11.7)	1 (ref)	.	1 (ref)	.
3–4	673	607 (90.2)	66 (9.8)	0.820 (0.569, 1.182)	0.287	0.766 (0.528, 1.109)	0.158
5–6	281	249 (88.6)	32 (11.4)	0.984 (0.785, 1.234)	0.891	.	.
≥ 7	117	107 (91.5)	10 (8.7)	0.890 (0.705, 1.123)	0.327	.	.
Severity with progressive COVID-19							
No	766	689 (89.9)	77 (10.1)	1 (ref)	.	1 (ref)	.
Yes	843	749 (88.8)	94 (11.6)	1.123 (0.817, 1.544)	0.475	1.031 (0.745, 1.426)	0.855
Antiviral treatment							
No	601	551 (91.7)	50 (8.3)	1 (ref)	.	1 (ref)	.
Yes	1,008	887 (88.0)	121 (12.0)	1.503 (1.063, 2.125)	0.021	1.503 (1.063, 2.126)	0.021
Immunosuppressant therapy							
No	1,031	920 (89.2)	111 (10.8)	1 (ref)	.	1 (ref)	.
Yes	578	518 (89.6)	60 (10.4)	0.960 (0.689, 1.338)	0.810	0.908 (0.647, 1.275)	0.577
Mechanical ventilation							
No	1,559	1,392 (89.3)	167 (10.7)	1 (ref)	.	1 (ref)	.
Yes	50	46 (92.0)	4 (8.0)	0.725 (0.258, 2.039)	0.542	0.724 (0.257, 2.041)	0.541

^a^We produced results using univariate and multivariate logistic regression models, with multivariate analyses using forward stepwise regression analysis. Categorical variables were expressed as frequencies (percentages). OR: odds ratio, CI: confidence interval, LC: long COVID.

**Table 5:** Logistic regression models to evaluate the risk factors for persistent LC vs. resolved LC.

Characteristics ^a^	Total	Resolved LC	Persistent LC	OR (95% CI)	p value	aOR (95% CI)	P value
Overall	313	192 (61.3)	121 (38.7)	.	.	.	.
Age (years)							
18–49	39	30 (76.9)	9 (23.1)	1 (ref)	.	1 (ref)	.
≥ 50	274	162 (59.1)	112 (40.9)	2.305 (1.053, 5.042)	0.037	1.747 (0.727, 4.199)	0.051
Gender							
Male	157	86 (54.8)	71 (45.2)	1 (ref)	.	1 (ref)	.
Female	156	106 (67.9)	50 (32.1)	0.571 (0.361, 0.905)	0.017	0.713 (0.409, 1.242)	0.074
Occupation							
Non-farmer	202	123 (60.9)	79 (39.1)	1 (ref)	.	1 (ref)	.
Farmer	111	69 (62.2)	42 (37.8)	1.055 (0.655, 1.699)	0.825	.	.
Vaccination							
Unvaccinated or one dose	72	40 (55.6)	32 (44.4)	1 (ref)	.	1 (ref)	.
Two or more doses	241	152 (63.1)	89 (36.9)	0.732 (0.429, 1.248)	0.251	.	.
Number of comorbidities							
0	13	11 (84.6)	2 (15.4)	1 (ref)	.	1 (ref)	.
≥ 1	300	181 (60.3)	119 (39.7)	3.616 (0.787, 16.604)	0.098	.	.
BMI (kg/m^2^)							
< 18.5	23	14 (60.9)	9 (39.1)	1.165 (0.466, 2.915)	0.742	.	.
18.5–23.9	121	78 (64.5)	43 (35.5)	1 (ref)	.	1 (ref)	.
24–27.9	123	73 (59.3)	50 (40.7)	1.242 (0.740, 2.085)	0.411	.	.
≥ 28	46	27 (58.7)	19 (41.3)	1.130 (0.798, 1.599)	0.491	1.184 (0.829, 1.690)	0.354
Smoke							
Never smoker	249	158 (63.5)	91 (36.5)	1 (ref)	.	1 (ref)	.
Current/Previous smoke	64	34 (56.7)	30 (43.3)	1.532 (0.880, 2.668)	0.132	.	.
Number of acute symptoms							
≤ 2	91	62 (68.1)	29 (31.9)	1 (ref)	.	1 (ref)	.
3–4	130	76 (58.5)	54 (41.5)	1.519 (0.866, 2.665)	0.145	1.476 (0.833, 2.615)	0.183
5–6	61	39 (63.9)	22 (36.1)	1.098 (0.780, 1.546)	0.591	.	.
≥ 7	31	15 (48.4)	16 (51.6)	1.316 (0.998, 1.736)	0.052	1.226 (0.905, 1.662)	0.188
Severity with progressive COVID-19							
No	119	79 (66.4)	40 (33.6)	1 (ref)	.	1 (ref)	.
Yes	194	113 (58.2)	81 (41.8)	1.416 (0.880, 2.278)	0.152	.	.
Antiviral treatment							
No	101	63 (62.4)	38 (37.6)	1 (ref)	.	1 (ref)	.
Yes	212	129 (60.8)	83 (39.2)	1.067 (0.655, 1.738)	0.795	.	.
Immunosuppressant therapy							
No	208	136 (65.4)	72 (34.6)	1 (ref)	.	1 (ref)	.
Yes	105	56 (53.3)	49 (36.7)	1.653 (1.025, 2.666)	0.039	1.513 (0.930, 2.463)	0.095
Mechanical ventilation							
No	304	187 (61.5)	117 (38.5)	1 (ref)	.	1 (ref)	.
Yes	9	5 (55.6)	4 (44.4)	1.279 (0.337, 4.858)	0.718	.	.

^a^ We produced results using univariate and multivariate logistic regression models, with multivariate analyses using forward stepwise regression analysis. Categorical variables were expressed as frequencies (percentages). OR: odds ratio, CI: confidence interval, LC: long COVID.

## Discussion

In this retrospective longitudinal cohort study, we systematically assessed the dynamic trajectory of LC in hospitalized patients with Omicron infection over a median follow-up of approximately 2 years after discharge. The key findings are threefold. First, the overall prevalence of LC at the second follow-up (15.2%) did not significantly decline from the first follow-up (16.3%), indicating a persistent symptom burden over time. Second, we identified two distinct trajectories [persistent LC (6.3%) and new-onset (8.9%) LC], highlighting that symptom emergence is not limited to the early post-infection period. Third, severe acute infection was associated with persistent LC, while new-onset LC was associated with different factors, suggesting potentially distinct underlying mechanisms. Together, these findings add to the current knowledge of the dynamics of health outcomes following COVID-19, particularly in the Omicron era and in an understudied Asian population.

At the second follow-up, the most common symptoms were muscle weakness, sleep difficulties, fatigue, cough, and dyspnea at rest. Muscle weakness, sleep difficulties, and cough showed significant increasing trends, whereas fatigue and dyspnea at rest remained stable. Post-COVID-19 fatigue resembles post-infectious fatigue syndromes documented for other infections (40), including SARS-CoV-1 (41) and Ebola virus (42), with SARS-CoV-1 fatigue lasting up to 4 years (43). In this study, a majority of the patients had dyspnea at rest at the second follow-up, consistent with a prior study (44) reporting a significant increase in the prevalence of dyspnea over time. The prevalence of LC at the second follow-up (15.2%) did not differ significantly from that at the first follow-up (16.3%), which aligns with previous longitudinal cohorts (14, 45). In particular, Huang et al. (14, 45) found no significant change in the prevalence of LC among original-strain survivors between year 1 and year 2 (49.0% vs. 55.0%). The results suggest that, although Omicron-infected patients have the lower prevalence of LC than original-strain patients, LC remains a long-term process. Severe COVID-19 patients were more likely to have persistent LC (7.8% vs. 4.5%, *p* = 0.003) but less likely to be asymptomatic (72.2% vs. 77.9%, *p* = 0.005) than non-severe patients. This finding is consistent with a previous original-strain study (31) in which severe cases also showed higher persistent symptoms (17.6% vs. 9.9%, *p* < 0.001) and lower asymptomatic rates (41.0% vs. 51.9%, *p* < 0.001). These findings indicate that severe acute illness predisposes patients to persistent LC. The 8.9% new-onset LC rate warrants careful clinical interpretation. This trajectory is fully consistent with the WHO definition, which states that LC symptoms “may be new onset following initial recovery…or persist…and may fluctuate or relapse” (7). It is further validated by the NIH RECOVER cohort (*n* = 3,659, 99.6% Omicron), in which 14.0% of participants did not meet LC criteria at 3 months but had increasing symptoms by 15 months—a pattern the authors attributed to “distinct pathophysiologic features” (32). The CHASING COVID cohort similarly defined new-onset LC symptoms not reported pre-infection and showed an elevated risk persisting up to 12 months (33). The 8.9% proportion in this study is comparable to RECOVER’s 14%, suggesting that this trajectory is not unique to the population but may be a general feature of post-Omicron recovery, possibly reflecting intermittent expression, gradual emergence of subthreshold symptoms, or true new-onset manifestations after a period of recovery. Notably, severe acute infection was associated with persistent LC but not new-onset LC, implying that these two trajectories may involve partially distinct underlying mechanisms.

The pathophysiological mechanisms underlying LC remain incompletely understood, with emerging evidence implicating multiple interwoven pathways including immune dysregulation, chronic inflammation, microvascular dysfunction, mitochondrial impairment, and persistent viral reservoirs (46–52). A detailed mechanistic exploration is beyond the scope of this descriptive study. Given this complexity, individual susceptibility to persistent symptoms may still be influenced by a combination of biological, clinical, and demographic factors. In this study, several factors associated with new-onset LC were identified. The observed association between antiviral treatment and new-onset LC should be interpreted with particular caution. Antiviral agents were preferentially administered to patients with higher disease severity or underlying risk factors (53). Although the study was adjusted for severity and comorbidities in multivariable models, confounding by indication cannot be fully excluded (54). Thus, the findings of this study should not be interpreted as evidence that antiviral treatment increases the risk of new-onset LC; rather, they reflect a subgroup with more complex recovery trajectories (55). Similarly, the apparently protective association of having one or more comorbidities with lower susceptibility to new-onset LC is counterintuitive and warrants careful interpretation. This finding may reflect survival bias, as patients with comorbidities who survived to follow-up may have been inherently healthier or received closer medical surveillance. Selection bias and model instability due to wide CIs and small subgroup sample sizes cannot be ruled out. Moreover, patients who are farmers by occupation had a significantly higher susceptibility to new-onset LC. This finding has not been widely reported and may reflect multiple interconnected factors: (1) lower health literacy and reduced access to timely medical follow-up; (2) sustained physical workload during the recovery period, potentially exacerbating fatigue and muscle weakness; (3) socioeconomic barriers to nutritional support and rehabilitation; and (4) potential occupational exposure to environmental dust or pesticides that could interact with post-inflammatory pulmonary vulnerability (56–58). Future studies should incorporate socioeconomic and occupational health data to disentangle these pathways. Female sex was associated with increased new-onset LC susceptibility, possibly due to X-linked immune gene effects (59), estrogen modulation of inflammatory responses (60), and a higher propensity to report symptoms (61). Low BMI (< 18.5 kg/m^2^) may indicate underlying frailty, malnutrition, or reduced metabolic reserve, which could impair viral clearance and tissue repair, increasing susceptibility to new-onset symptom emergence (62). However, Yang et al. (31) indicated that patients with pre-existing cerebrovascular disease had more coexisting disorders of other organ systems, raising the possibility that diseases other than COVID-19 may have been associated with new-onset symptoms. Therefore, it is difficult to determine whether new-onset symptoms are completely attributable to LC. The presence of patients with new-onset symptoms raises important questions about causality and the mechanisms that could result in symptom development at least three months after acute illness. In this study, new-onset symptoms may include patients who had a symptom that was mild at the first follow-up but moderate or severe at the second follow-up. The reclassification of patients into the new-onset group may also be influenced by the fluctuating nature of symptom severity. For instance, patients might have had a mild symptom at the first follow-up (below the threshold for reporting) that worsened to a moderate or severe level by the second follow-up due to triggers such as physical overexertion or seasonal illness. Alternatively, patients may have experienced symptom relapse after a period of near-complete recovery. This finding highlights the inherent complexity of defining LC trajectories and underscores the need for more frequent, real-time assessments in future studies to capture these dynamic changes accurately. Moreover, given the exploratory nature of subgroup and symptom-level analyses, these findings should be interpreted with caution and considered hypothesis-generating. Independent cohort studies are needed to confirm the observed association.

Several methodological limitations should be noted. First, the symptom-based telephone assessment did not include validated measures of symptom severity, functional impairment, or health-related quality of life. Consequently, the study cannot distinguish clinically significant LC that limits daily activities from mild, transient symptoms, which substantially constrains the clinical interpretation of the reported trajectories (35). Second, selection bias may have been introduced. The study exclusively included hospitalized COVID-19 patients from Changzhi, which may limit generalizability. Moreover, telephone-based follow-up may have under-represented patients with severe cognitive or functional impairments, as baseline data were collected only from patients who consented to participate. Due to data limitations, this study was unable to compare the characteristics of excluded patients (e.g., those who died, refused, or were unable to respond) with those of participants. While the likely direction of bias is toward the underestimation of LC prevalence, the impact of risk factor associations remains uncertain. This limitation is shared by most large-scale LC cohorts (29, 32–34, 37). Third, symptom attribution remains inherently challenging. The definition of new-onset LC relied on binary (presence/absence) symptom reporting at two time points, which cannot capture within-individual fluctuation or distinguish true new-onset from worsening of previously subthreshold symptoms—a limitation inherent to questionnaire-based longitudinal studies and shared by major cohorts, including RECOVER (32) and CHASING COVID (33). The study acknowledges that some symptoms assessed are non-specific and may be attributable to underlying comorbidities, intercurrent illness, or other causes (23–26). Although this study systematically inquired whether patients had experienced each symptom prior to infection and made efforts to distinguish new symptoms from preexisting conditions through detailed questioning, the retrospective nature and lack of documented pre-infection baseline data prevented objective verification. Furthermore, asymptomatic or untested reinfections during the prolonged follow-up could not be completely ruled out. Fourth, the study did not assess the association between clinical biomarkers during the acute phase of hospitalization and LC. Future studies should incorporate more frequent assessments, quantitative severity scales, in-person evaluations, objective biomarkers, and control groups of uninfected individuals to strengthen causal attribution, identify early risk indicators, and explore potential therapeutic targets.

In summary, this study reveals that Omicron-infected patients report a persistent burden of symptoms consistent with WHO-defined LC, with distinct trajectories of persistent (6.3%) and new-onset (8.9%) conditions extending beyond 2 years after discharge. Antiviral treatment, female sex, farmer by occupation, and BMI < 18.5 kg/m^2^ were associated with increased new-onset LC susceptibility, whereas having one or more complications was associated with decreased susceptibility. While the non-specific nature of these symptoms and the absence of functional impairment assessment preclude definitive etiological attribution, these findings provide valuable empirical evidence on the dynamic long-term health outcomes in Omicron-infected patients. Given these inherent limitations, the results are best interpreted as a descriptive foundation for future hypothesis-driven research, underscoring the urgency for prospective studies incorporating in-person assessments, objective biomarkers, and appropriate control groups to further elucidate the trajectory and mechanisms of LC.

## Data Availability

The original contributions presented in the study are included in the article/Supplementary material, further inquiries can be directed to the corresponding author/s.
