# Supplementary material for: Longitudinal trajectories of long COVID among hospitalized patients with omicron infection in Changzhi, China
Source: Front Med (Lausanne). 2026 Jul 28;13:1867430. doi: 10.3389/fmed.2026.1867430 (PMC13458740; doi:10.3389/fmed.2026.1867430)
Supplement: Supplementary file 1 [file Table_2.docx]

**S2 Table.** **Risk Factors associated with New-onset LC** **when the second follow-up was conducted at 375-660 days (17.0 [14.0, 20.1] months) during Omicron period**

| **Characteristics** ^a^ | **Total** | **No symptoms** | **New-onset LC** | **OR (95% CI)** | ***P* value** | ***aOR (95% CI)*** | ***P* value** |
| --- | --- | --- | --- | --- | --- | --- | --- |
| **Overall** | 313 | 280 (89.5) | 33 (10.5) | .. | *..* | .. | *..* |
| **Age (years)** | | | | | | | |
| 18-49 | 50 | 45 (90.0) | 5 (10.0) | 1 (ref) | .. | 1 (ref) | .. |
| ≥ 50 | 263 | 235 (89.4) | 28 (10.6) | 1.072 (0.393, 2.925) | 0.892 | 0.889 (0.296, 2.666) | 0.834 |
| **Gender** | | | | | | | |
| Male | 153 | 142 (92.8) | 11 (7.2) | 1 (ref) | .. | 1 (ref) | .. |
| Female | 160 | 138 (86.3) | 22 (13.7) | 2.058 (0.962, 4.404) | 0.063 | **3.729 (1.145, 12.147)** | 0.029 |
| **Occupation** | | | | | | | |
| Non-farmer | 224 | 211 (94.2) | 13 (5.8) | 1 (ref) | .. | 1 (ref) | .. |
| Farmer | 89 | 69 (77.5) | 20 (22.5) | **4.695 (2.222, 10.000)** | < 0.001 | **4.695 (2.188, 10.101)** | < 0.001 |
| **Vaccination** | | | | | | | |
| Unvaccinated or one dose | 88 | 77 (87.5) | 11 (12.5) | 1 (ref) | .. | 1 (ref) | .. |
| Two or more doses | 225 | 203 (90.2) | 22 (9.8) | 0.759 (0.351, 1.638) | 0.482 | 0.798 (0.355, 1.795) | 0.585 |
| **Number of comorbidities** | | | | | | | |
| 0 | 18 | 15 (83.3) | 3 (16.7) | 1 (ref) | .. | 1 (ref) | .. |
| ≥ 1 | 295 | 265 (89.8) | 30 (10.2) | 0.566 (0.155, 2.068) | 0.389 | 0.449 (0.110, 1.838) | 0.265 |
| **BMI (kg/m^2^)** | | | | | | | |
| < 18.5 | 21 | 16 (76.2) | 5 (23.8) | **3.876 (1.227, 12.821)** | 0.026 | **5.291 (1.115, 25.000)** | 0.036 |
| 18.5-23.9 | 134 | 124 (92.5) | 10 (7.5) | 1 (ref) | .. | 1 (ref) | .. |
| 24-27.9 | 111 | 97 (87.4) | 14 (12.6) | 1.790 (0.762, 4.204) | 0.182 | 2.031 (0.832, 4.959) | 0.120 |
| ≥ 28 | 47 | 43 (91.5) | 4 (8.5) | 1.074 (0.586, 1.967) | 0.817 | 1.104 (0.586, 2.081) | 0.759 |
| **Smoke** | | | | | | | |
| Never smoker | 222 | 198 (89.2) | 24 (10.8) | 1 (ref) | .. | 1 (ref) | .. |
| Current/Previous smoke | 91 | 82 (90.1) | 9 (9.9) | 0.905 (0.404, 2.032) | 0.810 | 2.502 (0.706, 8.862) | 0.155 |
| **Number of acute symptoms** | | | | | | | |
| ≤ 2 | 138 | 119 (86.2) | 19 (13.8) | 1 (ref) | .. | 1 (ref) | .. |
| 3-4 | 107 | 99 (92.5) | 8 (7.5) | 0.506 (0.212, 1.206) | 0.124 | 0.623 (0.253, 1.534) | 0.303 |
| 5-6 | 46 | 42 (91.3) | 4 (8.7) | 0.772 (0.438, 1.362) | 0.372 | 0.815 (0.443, 1.497) | 0.509 |
| ≥ 7 | 22 | 20 (90.9) | 2 (9.1) | 0.856 (0.513, 1.426) | 0.549 | 0.888 (0.527, 1.495) | 0.654 |
| **Severity with progressive COVID-19** | | | | | | | |
| No | 157 | 144 (91.7) | 13 (8.3) | 1 (ref) | .. | 1 (ref) | .. |
| Yes | 156 | 136 (87.2) | 20 (12.8) | 1.629 (0.780, 3.402) | 0.194 | 1.169 (0.530, 2.577) | 0.698 |
| **Antiviral treatment** | | | | | | | |
| No | 114 | 106 (93.0) | 8 (7.0) | 1 (ref) | .. | 1 (ref) | .. |
| Yes | 199 | 174 (87.4) | 25 (12.6) | 1.904 (0.828, 4.374) | 0.129 | 1.771 (0.748, 4.194) | 0.194 |
| **Immunosuppressant therapy** | | | | | | | |
| No | 212 | 187 (88.2) | 25 (11.8) | 1 (ref) | .. | 1 (ref) | .. |
| Yes | 101 | 93 (92.1) | 8 (7.9) | 0.643 (0.279, 1.482) | 0.300 | 0.616 (0.257, 1.475) | 0.277 |
| **Mechanical ventilation** | | | | | | | |
| No | 302 | 269 (89.1) | 33 (10.9) | 1 (ref) | .. | 1 (ref) | .. |
| Yes | 11 | 11 (100.0) | 0 (0.0) | .. | 0.999 | .. | 0.999 |

^a^ We produced results using univariate and multivariate logistic regression models, with multivariate analyses using forward stepwise regression analysis. Categorical variables were expressed as frequencies (percentages). OR: odds ratio, CI: confidence interval, LC: long COVID
