# Supplementary material for: Longitudinal trajectories of long COVID among hospitalized patients with omicron infection in Changzhi, China
Source: Front Med (Lausanne). 2026 Jul 28;13:1867430. doi: 10.3389/fmed.2026.1867430 (PMC13458740; doi:10.3389/fmed.2026.1867430)
Supplement: Supplementary file 2 [file Table_3.docx]

**S3 Table.** **Risk Factors associated with New-onset LC when the second follows-up was conducted 638-826 days (23.7 [23.3, 24.1] months) during Omicron period**

| **Characteristics** ^a^ | **Total** | **No symptoms** | **New-onset LC** | **OR (95% CI)** | ***P* value** | ***aOR (95% CI)*** | ***P* value** |
| --- | --- | --- | --- | --- | --- | --- | --- |
| **Overall** | 633 | 573 (90.5) | 60 (9.5) | .. | *..* | .. | *..* |
| **Age (years)** | | | | | | | |
| 18-49 | 77 | 73 (94.8) | 4 (5.2) | 1 (ref) | .. | 1 (ref) | .. |
| ≥ 50 | 556 | 500 (89.9) | 56 (10.1) | 2.044 (0.720, 5.804) | 0.179 | 2.590 (0.897, 7.483) | 0.079 |
| **Gender** | | | | | | | |
| Male | 322 | 299 (92.9) | 23 (7.1) | 1 (ref) | .. | 1 (ref) | .. |
| Female | 311 | 274 (88.1) | 37 (11.9) | **1.755 (1.017, 3.029)** | 0.043 | **1.971 (1.132, 3.431)** | 0.017 |
| **Occupation** | | | | | | | |
| Non-farmer | 427 | 391 (91.6) | 36 (8.4) | 1 (ref) | .. | 1 (ref) | .. |
| Farmer | 206 | 182 (88.3) | 24 (11.7) | 1.433 (0.830, 2.469) | 0.197 | 1.284 (0.737, 2.232) | 0.378 |
| **Vaccination** | | | | | | | |
| Unvaccinated or one dose | 154 | 139 (90.3) | 15 (9.7) | 1 (ref) | .. | 1 (ref) | .. |
| Two or more doses | 479 | 434 (90.6) | 45 (9.4) | 0.961 (0.520, 1.777) | 0.899 | 1.010 (0.543, 1.878) | 0.976 |
| **Number of comorbidities** | | | | | | | |
| 0 | 30 | 29 (96.7) | 1 (3.3) | 1 (ref) | .. | 1 (ref) | .. |
| ≥ 1 | 603 | 544 (90.2) | 59 (9.8) | 3.145 (0.421, 23.509) | 0.264 | 2.622 (0.329, 20.908) | 0.363 |
| **BMI (kg/m2)** | | | | | | | |
| < 18.5 | 40 | 35 (87.5) | 5 (12.5) | 1.506 (0.536, 4.237) | 0.437 | 1.403 (0.495, 3.968) | 0.525 |
| 18.5-23.9 | 254 | 232 (91.3) | 22 (8.7) | 1 (ref) | .. | 1 (ref) | .. |
| 24-27.9 | 250 | 225 (90.0) | 25 (10.0) | 1.172 (0.642, 2.138) | 0.606 | .. | .. |
| ≥ 28 | 89 | 81 (91.0) | 8 (9.0) | 1.021 (0.668, 1.559) | 0.925 | 1.025 (0.669, 1.571) | 0.910 |
| **Smoke** | | | | | | | |
| Never smoker | 481 | 432 (89.8) | 49 (10.2) | 1 (ref) | .. | 1 (ref) | .. |
| Current/Previous smoke | 152 | 141 (92.8) | 11 (7.2) | 0.688 (0.348, 1.359) | 0.281 | 1.003 (0.442, 2.277) | 0.994 |
| **Number of acute symptoms** | | | | | | | |
| ≤ 2 | 185 | 166 (89.7) | 19 (10.3) | 1 (ref) | .. | 1 (ref) | .. |
| 3-4 | 269 | 240 (89.2) | 29 (10.8) | 1.056 (0.573, 1.946) | 0.862 | 1.066 (0.576, 1.974) | 0.839 |
| 5-6 | 117 | 109 (93.2) | 8 (6.8) | 0.801 (0.521, 1.231) | 0.312 | 0.826 (0.535, 1.276) | 0.389 |
| ≥ 7 | 62 | 58 (93.6) | 4 (6.4) | 0.845 (0.582, 1.226) | 0.375 | 0.866 (0.594, 1.260) | 0.451 |
| **Severity with progressive COVID-19** | | | | | | | |
| No | 292 | 262 (89.7) | 30 (10.3) | 1 (ref) | .. | 1 (ref) | .. |
| Yes | 341 | 311 (91.2) | 30 (8.8) | 0.842 (0.495, 1.434) | 0.528 | 0.829 (0.476, 1.445) | 0.509 |
| **Antiviral treatment** | | | | | | | |
| No | 187 | 169 (90.4) | 18 (9.6) | 1 (ref) | .. | 1 (ref) | .. |
| Yes | 446 | 404 (90.6) | 42 (9.4) | 0.976 (0.546, 1.744) | 0.935 | 0.940 (0.512, 1.725) | 0.841 |
| **Immunosuppressant therapy** | | | | | | | |
| No | 439 | 396 (90.2) | 43 (9.8) | 1 (ref) | .. | 1 (ref) | .. |
| Yes | 194 | 177 (91.2) | 17 (8.8) | 0.885 (0.491, 1.594) | 0.683 | 0.921 (0.498, 1.706) | 0.795 |
| **Mechanical ventilation** | | | | | | | |
| No | 611 | 553 (90.5) | 58 (9.5) | 1 (ref) | .. | 1 (ref) | .. |
| Yes | 22 | 20 (90.9) | 2 (9.1) | 0.953 (0.217, 4.182) | 0.950 | 0.994 (0.223, 4.426) | 0.994 |

^a^ We produced results using univariate and multivariate logistic regression models, with multivariate analyses using forward stepwise regression analysis. Categorical variables were expressed as frequencies (percentages). OR: odds ratio, CI: confidence interval, LC: long COVID
