# Supplementary material for: Longitudinal trajectories of long COVID among hospitalized patients with omicron infection in Changzhi, China
Source: Front Med (Lausanne). 2026 Jul 28;13:1867430. doi: 10.3389/fmed.2026.1867430 (PMC13458740; doi:10.3389/fmed.2026.1867430)
Supplement: Supplementary file 3 [file Table_4.docx]

**S4 Table.** **Risk Factors associated with New-onset LC when the second follows-up was conducted 827-907 days (28.6 [28.3, 28.9] months) during Omicron period**

| **Characteristics** ^a^ | **Total** | **No symptoms** | **New-onset LC** | **OR (95% CI)** | ***P* value** | ***aOR (95% CI)*** | ***P* value** |
| --- | --- | --- | --- | --- | --- | --- | --- |
| **Overall** | 663 | 585 (88.2) | 78 (11.8) | .. | *..* | .. | *..* |
| **Age (years)** | | | | | | | |
| 18-49 | 121 | 110 (90.9) | 11 (9.1) | 1 (ref) | .. | 1 (ref) | .. |
| ≥ 50 | 542 | 475 (87.6) | 67 (12.4) | 1.411 (0.721, 2.758) | 0.315 | 1.622 (0.680, 3.870) | 0.275 |
| **Gender** | | | | | | | |
| Male | 336 | 289 (86.0) | 47 (14.0) | 1 (ref) | .. | 1 (ref) | .. |
| Female | 327 | 296 (90.5) | 31 (9.5) | 0.644 (0.398, 1.042) | 0.073 | 0.637 (0.383, 1.061) | 0.083 |
| **Occupation** | | | | | | | |
| Non-farmer | 476 | 416 (87.4) | 60 (12.6) | 1 (ref) | .. | 1 (ref) | .. |
| Farmer | 187 | 169 (90.4) | 18 (9.6) | 0.739 (0.423, 1.289) | 0.285 | 0.873 (0.492, 1.548) | 0.641 |
| **Vaccination** | | | | | | | |
| Unvaccinated or one dose | 202 | 184 (91.1) | 18 (8.9) | 1 (ref) | .. | 1 (ref) | .. |
| Two or more doses | 461 | 401 (87.0) | 60 (13.0) | 1.530 (0.878, 2.664) | 0.133 | 1.631 (0.927, 2.868) | 0.089 |
| **Number of comorbidities** | | | | | | | |
| 0 | 43 | 36 (83.7) | 7 (16.3) | 1 (ref) | .. | 1 (ref) | .. |
| ≥ 1 | 620 | 549 (88.5) | 71 (11.5) | 0.665 (0.285, 1.551) | 0.345 | **0.329 (0.125, 0.865)** | 0.024 |
| **BMI (kg/m2)** | | | | | | | |
| < 18.5 | 38 | 33 (86.8) | 5 (13.2) | 1.065 (0.389, 2.924) | 0.902 | 1.129 (0.399, 3.195) | 0.820 |
| 18.5-23.9 | 265 | 232 (87.6) | 33 (12.4) | 1 (ref) | .. | 1 (ref) | .. |
| 24-27.9 | 260 | 232 (89.2) | 28 (10.8) | 0.848 (0.497, 1.449) | 0.548 | 0.891 (0.519, 1.530) | 0.676 |
| ≥ 28 | 100 | 88 (88.0) | 12 (12.0) | 0.979 (0.688, 1.393) | 0.907 | 0.925 (0.634, 1.348) | 0.685 |
| **Smoke** | | | | | | | |
| Never smoker | 552 | 489 (88.6) | 63 (11.4) | 1 (ref) | .. | 1 (ref) | .. |
| Current/Previous smoke | 111 | 96 (86.5) | 15 (13.5) | 1.213 (0.663, 2.219) | 0.531 | 0.971 (0.498, 1.894) | 0.932 |
| **Number of acute symptoms** | | | | | | | |
| ≤ 2 | 215 | 190 (88.4) | 25 (11.6) | 1 (ref) | .. | 1 (ref) | .. |
| 3-4 | 297 | 268 (90.2) | 29 (9.8) | 0.822 (0.467, 1.449) | 0.499 | 0.684 (0.374, 1.248) | 0.215 |
| 5-6 | 118 | 98 (83.1) | 20 (16.9) | 1.245 (0.906, 1.712) | 0.177 | 1.149 (0.829, 1.594) | 0.405 |
| ≥ 7 | 33 | 29 (87.9) | 4 (12.1) | 1.016 (0.698, 1.478) | 0.935 | 0.970 (0.659, 1.428) | 0.877 |
| **Severity with progressive COVID-19** | | | | | | | |
| No | 317 | 283 (89.3) | 34 (10.7) | 1 (ref) | .. | 1 (ref) | .. |
| Yes | 346 | 302 (87.3) | 44 (12.7) | 1.213 (0.753, 1.952) | 0.427 | 1.192 (0.712, 1.995) | 0.503 |
| **Antiviral treatment** | | | | | | | |
| No | 300 | 276 (92.0) | 24 (8.0) | 1 (ref) | .. | 1 (ref) | .. |
| Yes | 363 | 309 (85.1) | 54 (14.9) | **2.010 (1.210, 3.338)** | 0.007 | **2.435 (1.381, 4.292)** | 0.002 |
| **Immunosuppressant therapy** | | | | | | | |
| No | 380 | 337 (88.7) | 43 (11.3) | 1 (ref) | .. | 1 (ref) | .. |
| Yes | 283 | 248 (87.6) | 35 (12.4) | 1.106 (0.688, 1.779) | 0.678 | 0.930 (0.559, 1.548) | 0.779 |
| **Mechanical ventilation** | | | | | | | |
| No | 646 | 570 (88.2) | 76 (11.8) | 1 (ref) | .. | 1 (ref) | .. |
| Yes | 17 | 15 (88.2) | 2 (11.8) | 1.000 (0.224, 4.458) | 1.000 | 1.079 (0.237, 4.911) | 0.922 |

^a^ We produced results using univariate and multivariate logistic regression models, with multivariate analyses using forward stepwise regression analysis. Categorical variables were expressed as frequencies (percentages). OR: odds ratio, CI: confidence interval, LC: long COVID
