# Supplementary material for: Longitudinal trajectories of long COVID among hospitalized patients with omicron infection in Changzhi, China
Source: Front Med (Lausanne). 2026 Jul 28;13:1867430. doi: 10.3389/fmed.2026.1867430 (PMC13458740; doi:10.3389/fmed.2026.1867430)
Supplement: Supplementary file 4 [file table_1.docx]

**Table S1. Symptom Questionnaire for long COVID-19**

| 1. Have you experienced fatigue now? | |
| --- | --- |
| □ No (go Q2) | □ Yes (go Q1.1) |
| 1.1 Is it newly onset post COVID-19? | |
| □ No (go Q1.2) | □ Yes (go Q2) |
| 1.2 Is it worse than the status prior to COVID-19? | |
| □ No | □ Yes |
| 2. Have you experienced muscle weakness now? | |
| □ No (go Q3) | □ Yes (go Q2.1) |
| 2.1 Is it newly onset post COVID-19? | |
| □ No (go Q2.2) | □ Yes (go Q3) |
| 2.2 Is it worse than the status prior to COVID-19? | |
| □ No | □ Yes |
| 3. Have you experienced sleep difficulty now? | |
| □ No (go Q4) | □ Yes (go Q3.1) |
| 3.1 Is it newly onset post COVID-19? | |
| □ No (go Q3.2) | □ Yes (go Q4) |
| 3.2 Is it worse than the status prior to COVID-19? | |
| □ No | □ Yes |
| 4. Have you experienced hair loss now? | |
| □ No (go Q5) | □ Yes (go Q4.1) |
| 4.1 Is it newly onset post COVID-19? | |
| □ No (go Q4.2) | □ Yes (go Q5) |
| 4.2 Is it worse than the status prior to COVID-19? | |
| □ No | □ Yes |
| 5. Have you experienced smell disorder now? | |
| □ No (go Q6) | □ Yes (go Q5.1) |
| 5.1 Is it newly onset post COVID-19? | |
| □ No (go Q5.2) | □ Yes (go Q6) |
| 5.2 Is it worse than the status prior to COVID-19? | |
| □ No | □ Yes |
| 6. Have you experienced palpitations now? | |
| □ No (go Q7) | □ Yes (go Q6.1) |
| 6.1 Is it newly onset post COVID-19? | |
| □ No (go Q6.2) | □ Yes (go Q7) |
| 6.2 Is it worse than the status prior to COVID-19? | |
| □ No | □ Yes |
| 7. Have you experienced decreased appetite now? | |
| □ No (go Q8) | □ Yes (go Q7.1) |
| 7.1 Is it newly onset post COVID-19? | |
| □ No (go Q7.2) | □ Yes (go Q8) |
| 7.2 Is it worse than the status prior to COVID-19? | |
| □ No | □ Yes |
| 8. Have you experienced taste disorder now? | |
| □ No (go Q9) | □ Yes (go Q8.1) |
| 8.1 Is it newly onset post COVID-19? | |
| □ No (go Q8.2) | □ Yes (go Q9) |
| 8.2 Is it worse than the status prior to COVID-19? | |
| □ No | □ Yes |
| 9. Have you experienced dizziness now? | |
| □ No (go Q10) | □ Yes (go Q9.1) |
| 9.1 Is it newly onset post COVID-19? | |
| □ No (go Q9.2) | □ Yes (go Q10) |
| 9.2 Is it worse than the status prior to COVID-19? | |
| □ No | □ Yes |
| 10. Have you experienced nausea or vomiting now? | |
| □ No (go Q11) | □ Yes (go Q10.1) |
| 10.1 Is it newly onset post COVID-19? | |
| □ No (go Q10.2) | □ Yes (go Q11) |
| 10.2 Is it worse than the status prior to COVID-19? | |
| □ No | □ Yes |
| 11. Have you experienced chest pain now? | |
| □ No (go Q12) | □ Yes (go Q11.1) |
| 11.1 Is it newly onset post COVID-19? | |
| □ No (go Q11.2) | □ Yes (go Q12) |
| 11.2 Is it worse than the status prior to COVID-19? | |
| □ No | □ Yes |
| 12. Have you experienced sore throat or difficult to swallow now? | |
| □ No (go Q13) | □ Yes (go Q12.1) |
| 12.1 Is it newly onset post COVID-19? | |
| □ No (go Q12.2) | □ Yes (go Q13) |
| 12.2 Is it worse than the status prior to COVID-19? | |
| □ No | □ Yes |
| 13. Have you experienced skin rash now? | |
| □ No (go Q14) | □ Yes (go Q13.1) |
| 13.1 Is it newly onset post COVID-19? | |
| □ No (go Q13.2) | □ Yes (go Q14) |
| 13.2 Is it worse than the status prior to COVID-19? | |
| □ No | □ Yes |
| 14. Have you experienced myalgia now? | |
| □ No (go Q15) | □ Yes (go Q14.1) |
| 14.1 Is it newly onset post COVID-19? | |
| □ No (go Q14.2) | □ Yes (go Q15) |
| 14.2 Is it worse than the status prior to COVID-19? | |
| □ No | □ Yes |
| 15. Have you experienced headache now? | |
| □ No (go Q16) | □ Yes (go Q15.1) |
| 15.1 Is it newly onset post COVID-19? | |
| □ No (go Q15.2) | □ Yes (go Q16) |
| 15.2 Is it worse than the status prior to COVID-19? | |
| □ No | □ Yes |
| 16. Have you experienced cough now? | |
| □ No (go Q17) | □ Yes (go Q16.1) |
| 16.1 Is it newly onset post COVID-19? | |
| □ No (go Q16.2) | □ Yes (go Q17) |
| 16.2 Is it worse than the status prior to COVID-19? | |
| □ No | □ Yes |
| 17. Have you experienced joint pain now? |  |
| □ No | □ Yes (fill in the table below )  If other joints, please specify___________ |

| Joints | Hand | Foot | Wrist | Ankle | Jaw | Elbow | Shoulder | Neck | Hip | Knee |
| --- | --- | --- | --- | --- | --- | --- | --- | --- | --- | --- |
| Tenderness |  |  |  |  |  |  |  |  |  |  |
| Swollen |  |  |  |  |  |  |  |  |  |  |
| Pain scale (0-10) |  |  |  |  |  |  |  |  |  |  |
| Newly onset post COVID-19  (if no, please fill in the next line) | □ No  □ Yes | □ No  □ Yes | □ No  □ Yes | □ No  □ Yes | □ No  □ Yes | □ No  □ Yes | □ No  □ Yes | □ No  □ Yes | □ No  □ Yes | □ No  □ Yes |
| Worse than the status prior to  COVID-19 |  |  |  |  |  |  |  |  |  |  |

0 (No pain)	10 (Intolerable pain)

18. Other symptoms needed to be recorded_____________.
